# Effects of Small-Sided Games vs. Running-Based High-Intensity Interval Training on Physical Performance in Soccer Players: A Meta-Analytical Comparison

**DOI:** 10.3389/fphys.2021.642703

**Published:** 2021-03-01

**Authors:** Filipe Manuel Clemente, Rodrigo Ramirez-Campillo, José Afonso, Hugo Sarmento

**Affiliations:** ^1^Escola Superior Desporto e Lazer, Instituto Politécnico de Viana Do Castelo, Rua Escola Industrial e Comercial de Nun'Álvares, Viana Do Castelo, Portugal; ^2^Instituto de Telecomunicações, Delegação da Covilhã, Lisbon, Portugal; ^3^Human Performance Laboratory, Quality of Life and Wellness Research Group, Department of Physical Activity Sciences, Universidad de Los Lagos, Lord Cochrane, Osorno, Chile; ^4^Centro de Investigación en Fisiología del Ejercicio, Facultad de Ciencias, Universidad Mayor, Santiago, Chile; ^5^Centre for Research, Education, Innovation and Intervention in Sport, Faculty of Sport of the University of Porto, Porto, Portugal; ^6^Research Unit for Sport and Physical Activity (CIDAF), Faculty of Sport Sciences and Physical Education, University of Coimbra, Coimbra, Portugal

**Keywords:** football (soccer), performance, drill-based games, conditioned games, interval training, motor skills

## Abstract

**Objectives:** This systematic review with meta-analysis (SRMA) was conducted to compare the effects of SSG-based interventions vs. running-based HIIT interventions on soccer players' sprinting time (ST), vertical height jump (VJH), and change of direction time (CODt).

**Data Sources:** The data sources utilized were Web of Science, Scopus, SPORTDiscus, and PubMed.

**Results:** An electronic search yielded 650 articles, six of which were included in the present study. Between-group analysis found a significant favoring effect of HIIT-based over SSG-based training interventions for the improvement of linear sprinting time (ES = 0.42; *p* = 0.012). A within-group analysis revealed a significant favoring effect of HIIT-based training interventions for improving linear sprinting time (ES = 0.42; *p* = 0.008) and CODt (ES = 1.04; *p* = 0.005) despite a non-significant effect on VJH (ES = 0.47; *p* = 0.22).

**Conclusions:** The meta-analytical comparison revealed favoring the effect of running-based HIIT over SSG-based interventions in sprinting performance, although no significant differences were observed for jumping and CODt performance. The findings suggest that SSG-based programs should be supplemented by other training methods that benefit determinant capacities in soccer players.

## Highlights

- Significant better benefits of running-based HIIT vs. SSGs were found in sprinting performance.
- None of interventions (running-based HIIT and SSGs) presented significant benefits in vertical jump performance.
- The SSG had no meaningful benefits in any of the outcomes (sprinting performance, vertical jump performance and change-of-direction performance).

## Introduction

Small-sided games (SSGs) (also known as small-sided and conditioned games) are conditioned and adjusted forms of a soccer game that are used in a training context to achieve a specific tactical/technical objective while changing physiological, physical, and psychological demands (Davids et al., 2013; Bujalance-Moreno et al., 2019; Clemente et al., 2020). In the context of soccer, these game-based drills are often used across the training week (Jeong et al., 2011), and the scientific interest about the effects of these games has been exponentially growing since the first systematic review published on the topic (Hill-Haas et al., 2011).

SSGs can produce highly variable acute responses considering the management of task constraints associated with them (Davids et al., 2013). In brief, smaller formats (fewer players), larger pitch sizes (higher individual playing area per player), a limited number of ball touches, man-to-man marking, or the use of small-goals or ball possession drills tend to increase the heart rate responses, blood lactate concentrations, and/or rate of perceived exertion of players from different age groups and competitive levels (Hill-Haas et al., 2011; Sarmento et al., 2018; Bujalance-Moreno et al., 2019). In terms of physical demands, consistent findings suggest an increase of distances covered in high-speed zones in SSGs played on larger pitches (Clemente et al., 2019); removing goalkeepers also contributes to an increase in distances covered at different intensities (Sarmento et al., 2018; Bujalance-Moreno et al., 2019). However, findings about the physical demands of changing formats, limitations of ball touches, action restrictions, and tactical/strategic missions are inconsistent (Sarmento et al., 2018; Bujalance-Moreno et al., 2019). Additionally, due to the inconsistency of findings about SSGs' effects on physical demands, it is somewhat difficult to use SSGs to impose highly intense physical demands (e.g., high-speed running, sprinting) when compared to traditional running-based drills that involve high-intensity interval training (HIIT) (Clemente, 2020; Clemente et al., 2021).

Despite that, the capacity of SSGs developing more than one performance dimension led to increased use of these games for providing a physical and physiological stimulus on the players. This approach has led the amount of scientific evidence about the effects of SSG-based programs started to increase, especially in the last decade (Hammami et al., 2018). Among the available state-of-the-art about the influence of SSGs in physical adaptations, the vast majority of findings focuses on the effects on aerobic performance in which no significant differences with running-based HIIT was found (Kunz et al., 2019; Moran et al., 2019; Clemente et al., 2021). Possibly, one of the justifications for this is the high-intensity and intermittent physiological effort imposed by SSGs, combined with low within- and between-players variability in terms of heart rate responses (Hill-Haas et al., 2008a; Stevens et al., 2016; Aquino et al., 2019).

Despite a good body of knowledge represented by a systematic review and a meta-analysis (SRMA) (Kunz et al., 2019; Moran et al., 2019) comparing the effects of SSG-based and running-based HIIT programs on aerobic performance, systematization of the effects in other determinant fitness variables is lacking. Due to the intermittent nature of soccer games (Iaia et al., 2009; Buchheit and Laursen, 2013a), which consist of low-to-moderate demands (e.g., jogging and running) interspaced by high-intensity activity (e.g., high-intensity running or sprinting) and explosive actions (e.g., jumping, changing direction, accelerating, and decelerating), it is important to ensure a proper fitness status that covers all the needs (Stolen et al., 2005). Among other factors, an improvement in sprinting time (ST) and change of direction time (CODt) may ensure a quicker capacity to overcome the opposing team, while a good vertical height jump (VHJ) may help players to reach higher balls while providing information about lower limb explosive power (Redkva et al., 2018).

As far as we know, no dedicated SRMA exists that compares the effects of SSG-based and running-based HIIT programs in soccer on ST, CODt, and VHJ. A systematization of information and evidence will help coaches to identify the potential adaptations promoted by drill-based games on these neuromuscular-related capacities. It may also provide new insights to researchers who are interested in this topic. For those reasons, the purpose of this SRMA was to compare the effects of SSG-based and running-based HIIT interventions on soccer players' sprinting, vertical jumping, and CODt performance.

## Methods

The present SRMA followed the Cochrane Collaboration guidelines (Green and Higgins, 2005). The systematic review strategy was conducted according to PRISMA (Preferred Reporting Items for Systematic Reviews and Meta-analyses) guidelines (Moher et al., 2009). The protocol was registered with the International Platform of Registered Systematic Review and Meta-Analysis Protocols with the number INPLASY202080114 and the DOI number 10.37766/inplasy2020.8.0114.

### Information Sources

A comprehensive computerized search of the following electronic databases was performed: (i) Web of Science; (ii) Scopus; (iii) SPORTdiscus; and (iv) PubMed. The searching process for relevant publications had no restriction regarding year of publication and included articles retrieved until 27^th^ August 2020. The following search strings were employed: (“soccer” OR “football”) AND (“small-sided games” OR “drill-based games” OR “sided-games” OR “SSG” OR “conditioned games” OR “small-sided and conditioned games” OR “reduced games” OR “play formats”) AND (“sprint^*^” OR OR “velocity^*^” OR “vertical jump^*^” OR “jump^*^” OR “countermovement jump” OR “CMJ” OR “squat jump” OR “SJ” OR “drop jump” OR “DJ” OR “change of direction” OR “COD” OR “agility”).

The *a priori* inclusion criteria for this review were as follows: (i) parallel studies (SSG-based programs vs. running-based HIIT) conducted in soccer players with no restriction of age, sex or competitive level; (ii) isolated intervention programs (i.e., only SSG vs. only running-based HIIT—not combined forms) with no restrictions for duration; (iii) a pre-post outcome for physical fitness, including ST, VHJ, and CODt; (iv) original per-reviewed articles written in English that provided full-text. Studies were excluded on the basis that they: (i) were observational analytic designs; (ii) included other sports; (iii) used SSG or running-based HIIT combined with other training methods or between them (e.g., SSG + running based-HIIT); (iv) conducted in recreational soccer (e.g., healthy population but not soccer players) or physical education contexts; and (iv) were review articles, letters to the editor, errata, invited commentaries, or conference abstracts.

### Data Extraction

A data extraction sheet conceived in Microsoft Excel (Microsoft Corporation, Readmon, WA, USA) was made based on Cochrane Consumers and Communication Review Group's data extraction template (Group C. C. C. R., 2016). The sheet was used to assess inclusion requirements. The process was conducted by two of the authors (FC and HS). Any disagreement regarding study eligibility was resolved in a discussion between the authors. Full text articles excluded, with reasons, were recorded. All the records were stored in the sheet.

### Data Items

The outcomes chosen for this SRMA included ST, VHJ, and CODt. The linear ST(s) at different distances was collected, without including values of partial times and extracted only from linear sprinting tests. The VHJ (measured in cm) was usually assessed during a countermovement jump (CMJ) with or without arm swing, squat jump (SJ) or drop jump (DJ). The CODt was regularly measured at COD tests and the time for performing the test was collected. Additionally, the following information was extracted from the included studies: (i) number of participants (*n*), age (years), competitive level (if available), and sex; (ii) the SSGs format and pitch size (if available); (iii) period of intervention (number of weeks) and number of sessions per week (*n*/w); and (iv) regimen of intervention (work duration, work intensity, modality, relief duration, relief intensity, repetitions and series, between-set recovery).

### Assessment of Methodological Quality

The methodological index for non-randomized studies (MINORS) was used (Slim et al., 2003) to assess the parallel studies. Twelve items were analyzed, in which 0 represented cases of no report, 1 cases of report but inadequate, and 2 in cases of report and adequate. Two of the authors (FC and HS) independently scored the articles. Disagreements in the rating between both authors was resolved through discussion. Aiming to control the risk of bias between authors, the Kappa correlation test was used to analyze the agreement level for the included studies. An agreement level of *k* = 0.87 was obtained.

### Summary Measures

Meta-analysis was conducted in the case of at least three study groups provided baseline and follow-up data for the same measure (Moran et al., 2018; García-Hermoso et al., 2019; Skrede et al., 2019). Means and standard deviations for a measure (ST; VHJ; CODt) of pre-post SSG-based interventions were used to compute the Hedges's *g* effect size (ES). The inverse variance random-effects model for meta-analyses was used because it allocates a proportionate weight to trials based on the size of their individual standard errors (Deeks et al., 2008) and enables analysis while accounting for heterogeneity across studies (Kontopantelis et al., 2013). The ESs were presented alongside 95% confidence intervals (CIs) and interpreted using the following thresholds (Hopkins et al., 2009): <0.2, trivial; 0.2–0.6, small; >0.6–1.2, moderate; >1.2–2.0, large; >2.0–4.0, very large; >4.0, extremely large. All analyses were carried out using the Comprehensive Meta-Analysis program (version 2; Biostat, Englewood, NJ, USA).

### Synthesis of Results

To estimate the degree of heterogeneity between the included studies, the percentage of total variation across the studies was used to calculate the *I*^2^ statistic (Higgins, 2003). Low, moderate, and high levels of heterogeneity correspond to *I*^2^-values of <25, 25–75, and >75%, respectively (Higgins and Thompson, 2002; Higgins, 2003).

### Risk of Bias Across Studies

The extended Egger's test (Egger et al., 1997) was used to assess the risk of bias across the studies. In case of bias, a sensitivity analysis was conducted.

## Results

### Study Identification and Selection

**Figure 1:**
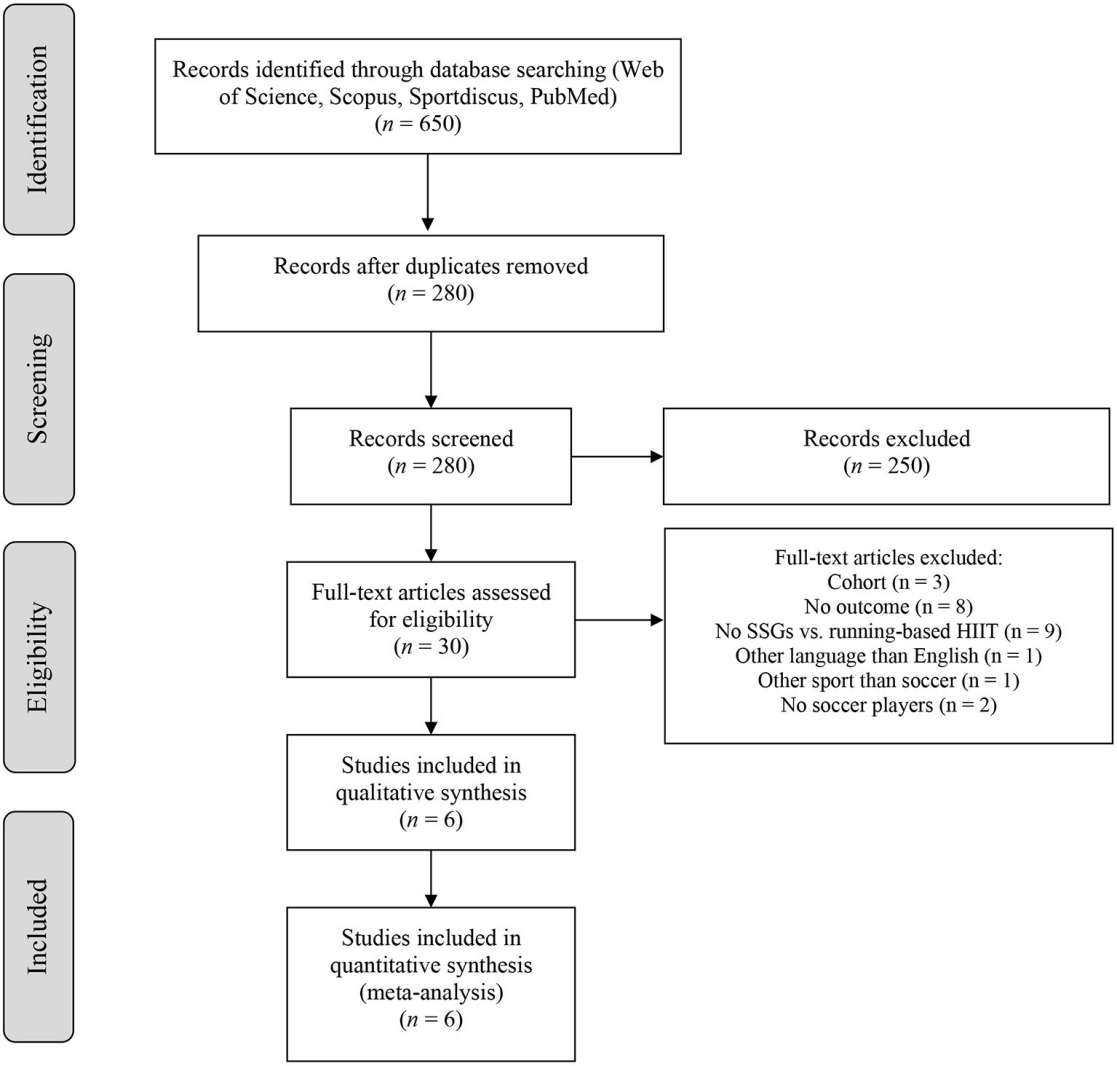
PRISMA flow diagram highlighting the selection process for the studies included in the systematic review and meta-analysis.

The searching of databases identified a total of 650 items. These studies were then exported to reference manager software (EndNote™ X9, Clarivate Analytics, Philadelphia, PA, USA). Duplicates (370 references) were subsequently removed either automatically or manually. The remaining 280 articles were screened for their relevance based on titles and abstracts, removing 250 studies. The full texts of the remaining 30 articles were examined diligently. After reading full texts, a further 24 studies were excluded due to a number of reasons (Figure 1). The six studies included in the meta-analysis provided mean and standard deviation for pre- and post-interventions data for at least one main outcome.

### Study Characteristics

The characteristics of the six studies included in the SRMA can be found in Table 1. Additionally, the details of the SSG-based and running-based HIIT programs can be found in Tables 2, 3, respectively. The included parallel studies involved 12 individual groups (6 SSG-based groups and 6 running-based HIIT groups) and 156 participants (*n* = 76 in SSG-based groups; *n* = 88 in running-based HIIT groups). The six studies were conducted in youth male soccer players. The shorter intervention lasted 4 weeks (Faude et al., 2014) and the longer 8 (Radziminski et al., 2013; Jastrzebski et al., 2014; Stojiljković et al., 2019). Moreover, the minimum number of sessions were 8 (Faude et al., 2014) and the maximum 16 (Radziminski et al., 2013; Jastrzebski et al., 2014; Stojiljković et al., 2019).

**Table 1:** Characteristics of the included studies and outcomes extracted.

**Study**	**Mean age (yo)**	**Sex**	**CL**	**Design**	**Outcomes**	**Tests used in the original studies**	**Measure extracted from the tests in the original studies**
Arcos et al. (2015)	15.5	Men	Y	Parallel	VHJ	VHJ: CMJ	VHJ: (cm)
Arslan et al. (2020)	14.2	Men	Y	Parallel	VHJ; ST; CODt	VHJ: CMJ	VHJ: (cm)
						ST: linear sprint 30-m	ST: time (s)
						CODt: ZAWOB	CODt: time (s)
Faude et al. (2014)	16.5	Men	Y	Parallel	VHJ; ST; CODt	VHJ: CMJ	VHJ: (cm)
						ST: linear sprint 30-m	ST: time (s)
						CODt: 20-m COD with 6 sharp turns	CODt: time (s)
Jastrzebski et al. (2014)	15.8	Men	Y	Parallel	ST	ST: linear sprint 30-m	ST: time (s)
Radziminski et al. (2013)	15.1	Men	Y	Parallel	ST	ST: linear sprint 30-m	ST: time (s)
Stojiljković et al. (2019)	15.6	Men	Y	Parallel	VHJ; ST; CODt	VHJ: CMJ	VHJ: (cm)
						ST: linear sprint 20-m	ST: time (s)
						CODt: Illinois test	CODt: time (s)

*N, number of participants in the study; Yo, years old; CL, competitive level; Y, youth; VHJ, vertical height jump; ST, sprinting time at linear trajectories; CODt, change-of-direction time; s, seconds; cm, centimeters; m, meters; ZAWOB, zigzag agility without the ball*.

**Table 2:** Characteristics of SSG-based programs in the included studies.

**Study**	**Duration (w)**	**d/w**	**Total sessions**	**SSG formats**	**SSG pitch dimension** **(length × width)**	**SSG area per player (m^**2**^)**	**Sets**	**Reps**	**Work duration**	**Work intensity**	**Relief duration**	**Relief intensity**
Arcos et al. (2015)	6	2	12	3 vs. 3 to 4 vs. 4	NR	85 m^2^	–	3	4 min	NR	3 min	Passive
Arslan et al. (2020)	5	2	10	2 vs. 2	20 × 15-m	75 m^2^	2	2	2.5–4.5 min	NR	2 min	Passive
Faude et al. (2014)	4	2	8	3 vs. 3 to 4 vs. 4	35 × 25-m to 40 × 30-m	145 to 150 m^2^	–	4	4 min	NR	4 min	Technical activities
Jastrzebski et al. (2014)	8	2	16	3 vs. 3	18 × 30-m	90 m^2^	–	7	3 min	NR	90 s	Active recovery
Radziminski et al. (2013)	8	2	16	3 vs. 3 or 3 vs. 3+1	18 × 30-m	77–90 m^2^	–	5	4 min	>90% HRmax	3 min	Light activity
Stojiljković et al. (2019)	8	2	16	3 vs. 3*	20 × 15-m	50 m^2^	–	NR	NR	NR	NR	NR
				4 vs. 4*	25 × 18-m	56 m^2^						

SSGs, small-sided games; W, weeks; d/w, days per week; NR, not reported; m, meters; s, seconds; min, minutes;

* *ball contacts restricted; GK, goalkeeper*.

**Table 3:** Characteristics of running-based HIIT programs in the included studies.

**Study**	**Duration (w)**	d/w^*^	**Total sessions**	Work duration^*^	**Work intensity**	**Relief duration**	**Relief intensity**	Sets^*^	Reps^*^	**Recovery between sets (duration)**	**Recovery between sets (intensity)**
Arcos et al. (2015)	6	2	12	4 min	90–95% HRmax	3 min	50–60% HRmax	–	3	–	–
Arslan et al. (2020)	5	2	10	15 s	90–95% V_IFT_	15 s	Passive	2	12–20	NR	NR
Faude et al. (2014)	4	2	8	15 s	140% IAT	15 s	NR	2	12–15	10 min	NR
Jastrzebski et al. (2014)	8	2	16	15 s	85–90% HRmax	15 s	NR	7	6	90 s	Active recovery
Radziminski et al. (2013)	8	2	16	4 min	>90% HRmax	3 min	Light activity	–	5	–	–
Stojiljković et al. (2019)	8	2	16	15 s	90–95% HRmax	15 s	NR	3–4	5–8	NR	NR

W, weeks; d/w, days per week; s, seconds; NR, not reported.

* *The range between the programs; min, minutes; m, meters; V_IFT_, maximal velocity at 30–15 IFT; IAT, individual anaerobic threshold; HRmax, maximal heart rate; Passive, passive recovery; s, seconds; min, minutes; m, meters*.

### Methodological Quality

In the case of parallel studies, 3 studies were scored between 16 and 17 points, and 3 studies between 18 and 19 points (Table 4).

**Table 4:** Methodological index for non-randomized studies (MINORS).

	N.^**°**^1^*^	**N.^**°**^2**	**N.^**°**^3**	**N.^**°**^4**	**N.^**°**^5**	**N.^**°**^6**	**N.^**°**^7**	**N.^**°**^8**	**N.^**°**^9**	**N.^**°**^10**	**N.^**°**^11**	**N.12**	Total^**^
Arcos et al. (2015)	2	1	2	2	0	2	2	0	1	2	2	2	18
Arslan et al. (2020)	2	1	2	2	0	2	2	0	1	2	2	2	18
Faude et al. (2014)	2	0	2	2	0	2	0	0	2	2	2	2	16
Jastrzebski et al. (2014)	2	1	2	2	0	2	0	0	1	2	2	2	16
Radziminski et al. (2013)	2	1	2	2	0	2	0	0	1	2	2	2	16
Stojiljković et al. (2019)	2	1	2	2	0	2	2	0	1	2	2	2	18

* MINORS scale items number; N.°1, A clearly study aimed; N.° 2, Inclusion of consecutive patients; N.° 3, Prospective collection of data; N.°4, Endpoints appropriate to the aim of the study; N.° 5, Unbiased assessment of the study endpoint; N.° 6, Follow-up period appropriate to the aim of the study; N.° 7, Loss to follow <5%; N.° 8, Prospective calculation of the study size; N.° 9, An adequate control group; N.°10, Contemporary groups; N.°11, Baseline equivalence of groups; N.°12, Adequate statistical analyses.

** *The total number of points from a possible maximal of 24*.

### SSG vs. Running-Based HIIT Interventions on Linear Sprinting Time

A summary of the included studies and results of ST reported before and after SSG-based and running-based HIIT interventions are provided in Table 5.

**Table 5:** Summary of the included studies and results of linear sprinting time before and after SSG-based and running-based HIIT intervention.

**Study**	**Intervention**	***N***	**Before**	**After**	**Before–After**
			**Mean ± SD**	**Mean ± SD**	**(Δ%)**
Arslan et al. (2020)	SSG	10	5.15 ± 0.32	4.81 ± 0.31	−6.6
Faude et al. (2014)	SSG	9	4.13 ± 0.13	4.13 ± 0.11	0.0
Jastrzebski et al. (2014)	SSG	11	4.61 ± 0.25	4.67 ± 0.25	1.3
Radziminski et al. (2013)	SSG	9	4.91 ± 0.29	4.89 ± 0.40	−0.4
Stojiljković et al. (2019)	SSG	30	3.27 ± 0.18	3.23 ± 0.14	−1.2
Arslan et al. (2020)	rbHIIT	10	5.00 ± 0.34	4.66 ± 0.29	−6.8
Faude et al. (2014)	rbHIIT	10	4.12 ± 0.13	4.09 ± 0.11	−0.7
Jastrzebski et al. (2014)	rbHIIT	11	4.66 ± 0.22	4.62 ± 0.22	−0.9
Radziminski et al. (2013)	rbHIIT	11	4.80 ± 0.28	4.77 ± 0.24	−0.6
Stojiljković et al. (2019)	rbHIIT	30	3.36 ± 0.21	3.22 ± 0.11	−4.2

*RCT, randomized-controlled trial; n, number of participants per group; SD, standard deviation; SSG, small-sided game; rbHIIT, running-based HIIT*.

**Figure 2:**
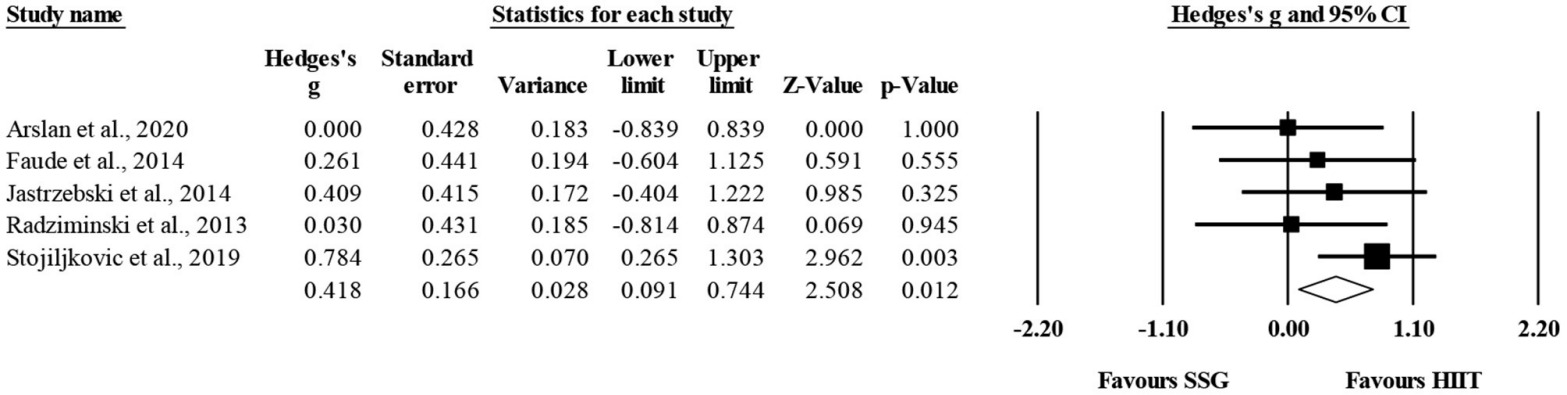
Forest plot of between-mode effect sizes (Hedges's g) with 95% confidence intervals (CIs) in sprinting time. SSG, small-sided games; HIIT, running-based high-intensity interval training.

Five studies provided data for linear sprinting time, involving five SSG-based and five HIIT-based groups (pooled *n* = 141). There was a significant favoring effect of HIIT-based over SSG-based training interventions for the improvement of linear sprinting time (ES = 0.42; 95% CI = 0.09 to 0.74; *p* = 0.012; *I*^2^ = 0.0%; Egger's test *p* = 0.025; Figure 2). The relative weight of each study ranged from 14.3 to 39.6% (the size of the plotted squares reflects the statistical weight of each study). The adjusted values (based on Duval and Tweedie's trim and fill method) indicated an ES = 0.57 (95% CI = 0.22 to 0.92) favoring HIIT-based over SSG-based training interventions.

**Figure 3:**
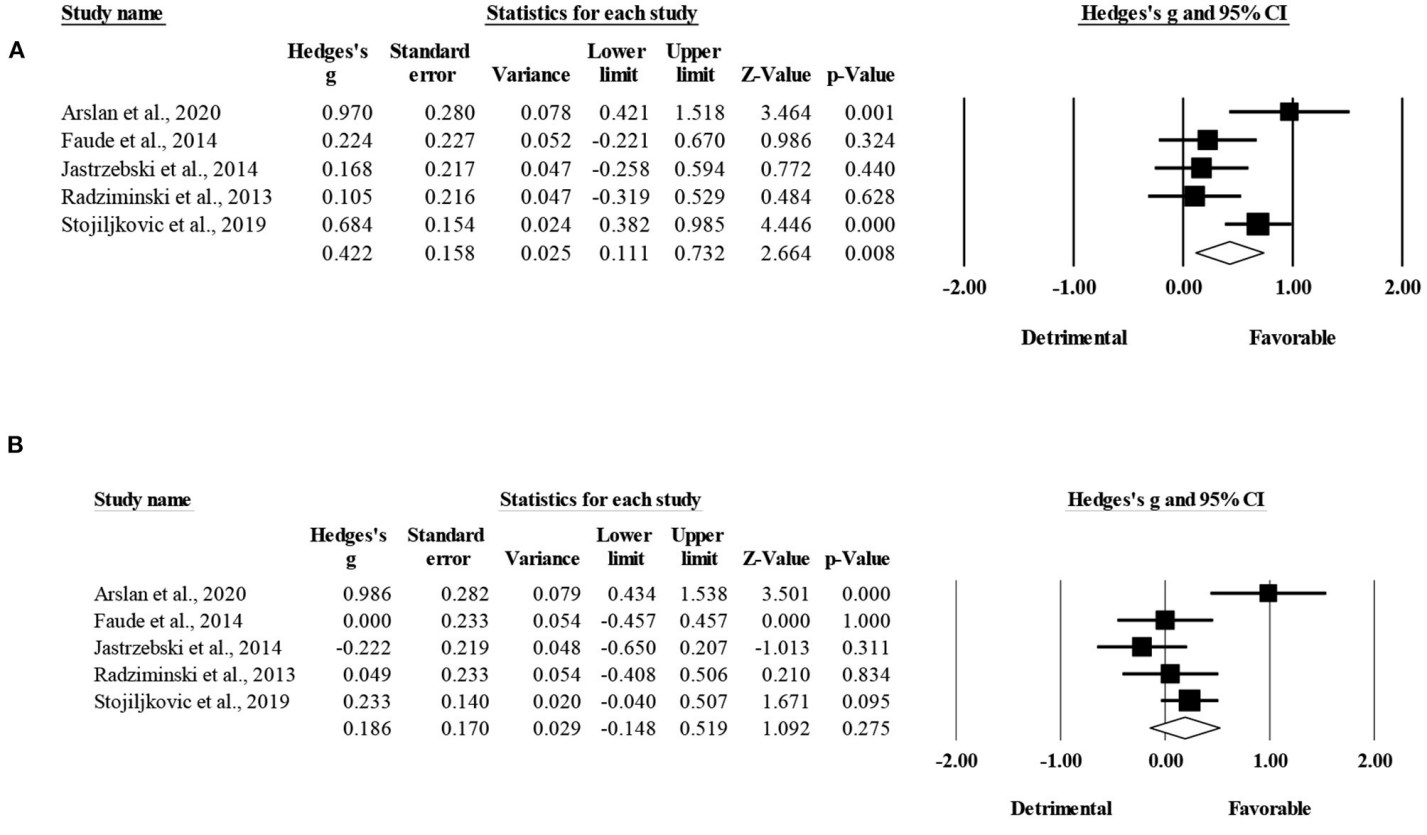
Forest plot of within-mode effect sizes (Hedges's g) with 95% confidence intervals (CIs) in sprinting time. **(A)** HIIT, running-based high-intensity interval training; **(B)** SSG, small-sided games.

The within-group analysis revealed a significant favoring effect of HIIT-based training interventions for the improvement of linear sprinting time (ES = 0.42; 95% CI = 0.11 to 0.73; *p* = 0.008; *I*^2^ = 63.5%; Egger's test *p* = 0.770; Figure 3A). The relative weight of each study ranged from 16.1 to 24.6%.

The within-group analysis revealed a non-significant effect of SSG-based training interventions on linear sprinting time (ES = 0.19; 95% CI = −0.15 to 0.52; *p* = 0.28; *I*^2^ = 68.3%; Egger's test *p* = 0.801; Figure 3B). The relative weight of each study in the analysis ranged from 16.5 to 25.0%.

### SSG vs. Running-Based HIIT Interventions on Vertical Height Jump

A summary of the included studies and results of VHJ reported before and after SSG-based and running-based HIIT interventions are provided in Table 6.

**Table 6:** Summary of the included studies and results of vertical height jump before and after SSG-based and running-based HIIT intervention.

**Study**	**Design**	***N***	**Before**	**After**	**Before–After**
			**Mean ± SD**	**Mean ± SD**	**(Δ%)**
Arcos et al. (2015)	SSG	7	42.7 ± 2.4	42.0 ± 2.8	−1.6
Arslan et al. (2020)	SSG	10	28.5 ± 2.5	31.3 ± 1.9	9.8
Faude et al. (2014)	SSG	9	38.1 ± 4.7	37.5 ± 4.6	−1.6
Stojiljković et al. (2019)	SSG	30	41.6 ± 4.8	42.6 ± 4.3	2.4
Arcos et al. (2015)	rbHIIT	8	42.8 ± 4.6	42.4 ± 4.8	−0.9
Arslan et al. (2020)	rbHIIT	10	28.2 ± 2.0	30.6 ± 1.8	8.5
Faude et al. (2014)	rbHIIT	10	38.5 ± 4.0	37.3 ± 4.0	−3.1
Stojiljković et al. (2019)	rbHIIT	30	39.8 ± 4.8	46.5 ± 6.5	16.8

*C, controlled; NC, non-controlled; n, number of participants per group; SD, standard deviation; SSG, small-sided game; rbHIIT, running-based HIIT*.

**Figure 4:**
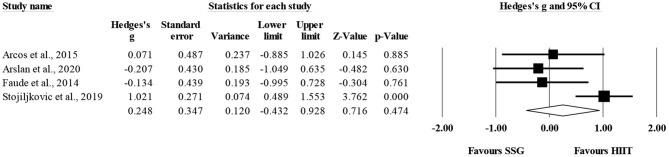
Forest plot of between-mode effect sizes (Hedges's g) with 95% confidence intervals (CIs) in vertical height jump. SSG, small-sided games; HIIT, running-based high-intensity interval training.

Four studies provided data for VJH, involving four SSG-based and four HIIT-based groups (pooled *n* = 114). There was no significant difference between HIIT-based compared to SSG-based training interventions on VJH changes (ES = 0.25; 95% CI = −0.43 to 0.93; *p* = 0.474; *I*^2^ = 67.1%; Egger's test *p* = 0.056; Figure 4). The relative weight of each study ranged from 21.7 to 30.8%.

**Figure 5:**
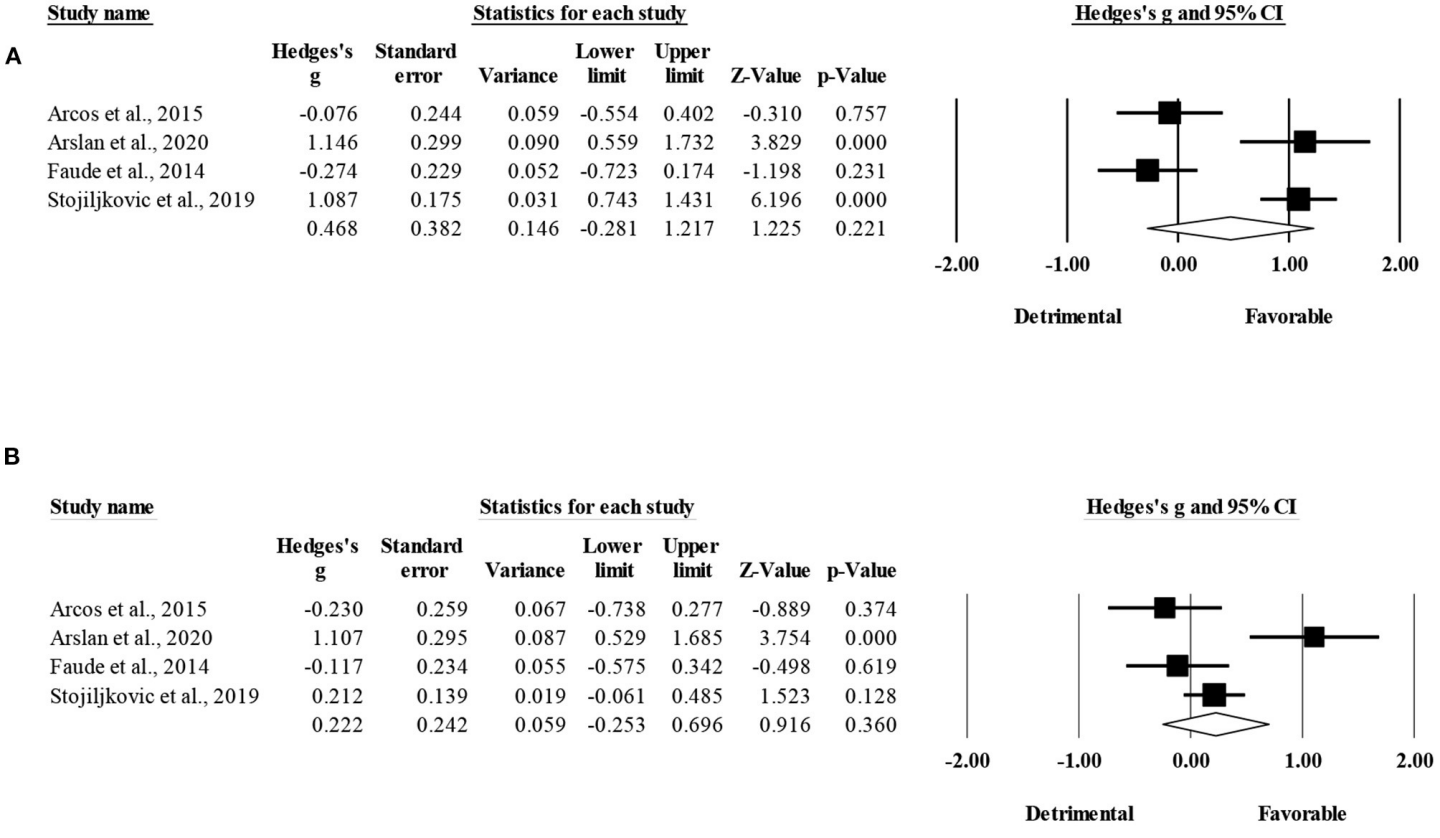
Forest plot of within-mode effect sizes (Hedges's g) with 95% confidence intervals (CIs) in vertical height jump. **(A)** HIIT, running-based high-intensity interval training; **(B)** SSG, small-sided games.

The within-group analysis revealed a non-significant effect of HIIT-based training interventions on VJH (ES = 0.47; 95% CI = −0.28 to 1.22; *p* = 0.22; *I*^2^ = 90.9%; Egger's test *p* = 0.706; Figure 5A). The relative weight of each study in the analysis ranged from 23.7 to 26.2%.

The within-group analysis revealed a non-significant effect of SSG-based training interventions on VJH (ES = 0.22; 95% CI = −0.25 to 0.70; *p* = 0.36; *I*^2^ = 78.6%; Egger's test *p* = 0.836; Figure 5B). The relative weight of each study in the analysis ranged from 22.0 to 29.4%.

### SSG vs. Running-Based HIIT Interventions on Change of Direction Time

A summary of the included studies and results of CODt reported before and after SSG-based and running-based HIIT interventions are provided in Table 7.

**Table 7:** Summary of the included studies and results of change-of-direction time before and after SSG-based and running-based HIIT intervention.

**Study**	**Design**	***N***	**Before**	**After**	**Before–After**
			**Mean ± SD**	**Mean ± SD**	**(Δ%)**
Arslan et al. (2020)	SSG	10	6.9 ± 0.2	6.7 ± 0.2	−2.9
Faude et al. (2014)	SSG	9	7.9 ± 0.2	7.9 ± 0.3	0
Stojiljković et al. (2019)	SSG	30	14.9 ± 0.4	15.1 ± 0.6	1.3
Arslan et al. (2020)	rbHIIT	10	7.1 ± 0.2	6.9 ± 0.2	−2.8
Faude et al. (2014)	rbHIIT	10	7.9 ± 0.3	7.7 ± 0.4	−2.5
Stojiljković et al. (2019)	rbHIIT	30	15.7 ± 0.5	14.9 ± 0.4	−5.1

*C, controlled; NC, non-controlled; n, number of participants per group; SD, standard deviation; SSG, small-sided game; rbHIIT, running-based HIIT*.

**Figure 6:**
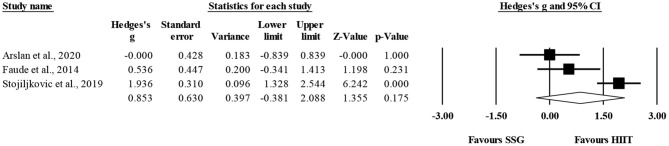
Forest plot of between-mode effect sizes (Hedges's g) with 95% confidence intervals (CIs) in change-of-direction. SSG, small-sided games; HIIT, running-based high-intensity interval training.

Three studies provided data for CODt, involving three SSG-based and three HIIT-based groups (pooled *n* = 99). There was no significant difference between HIIT-based compared to SSG-based training interventions on CODt changes (ES = 0.85; 95% CI = −0.38 to 2.09; *p* = 0.175; *I*^2^ = 87.1%; Egger's test *p* = 0.208; Figure 6). The relative weight of each study in the analysis ranged from 32.2 to 35.2%.

**Figure 7:**
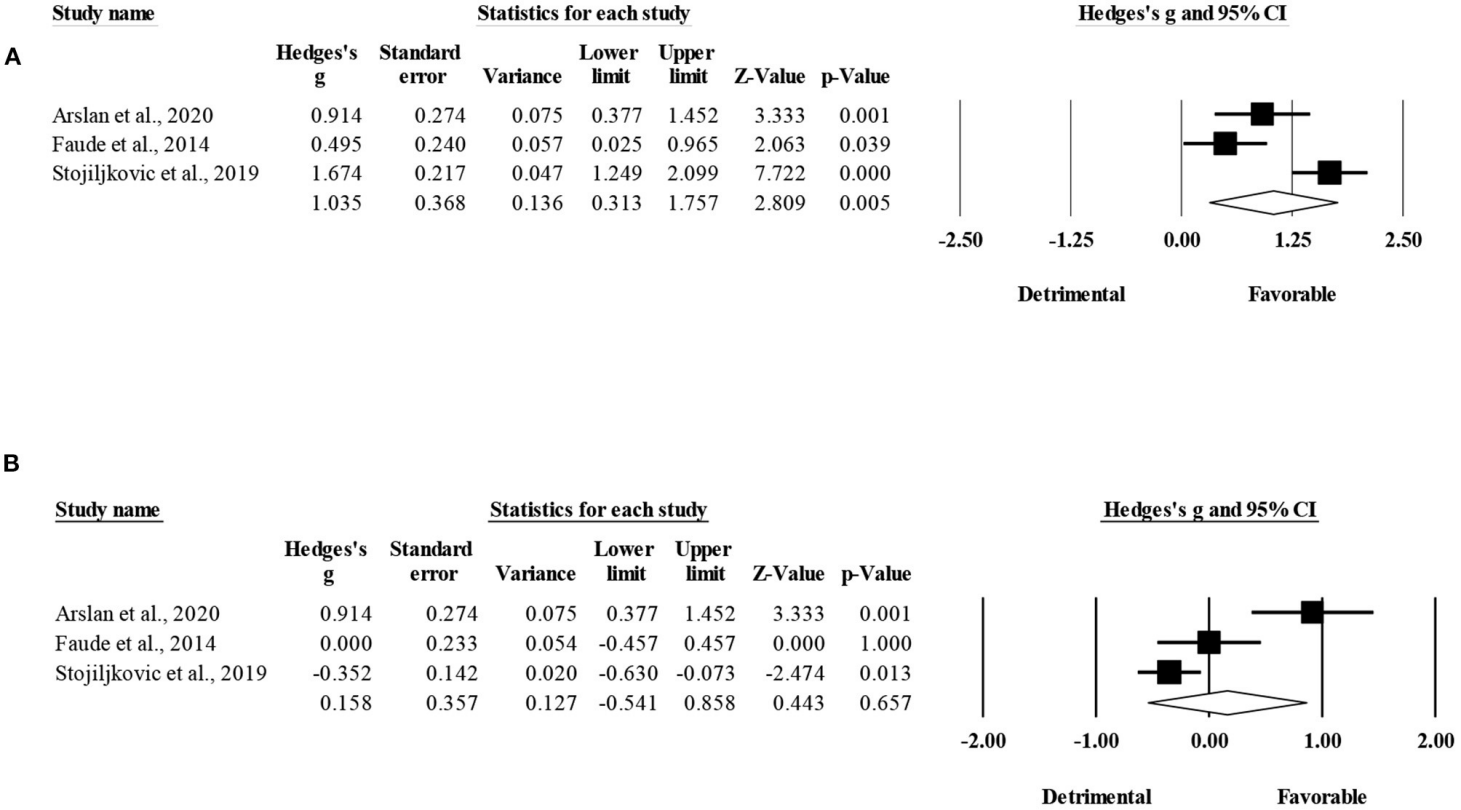
Forest plot of within-mode effect sizes (Hedges's g) with 95% confidence intervals (CIs) in change-of-direction. **(A)** HIIT, running-based high-intensity interval training; **(B)** SSG, small-sided games.

The within-group analysis revealed a significant favoring effect of HIIT-based training interventions for the improvement of CODt (ES = 1.04; 95% CI = 0.31 to 1.76; *p* = 0.005; *I*^2^ = 85.5%; Egger's test *p* = 0.590; Figure 7A). The relative weight of each study in the analysis ranged from 32.1 to 34.4%.

The within-group analysis revealed a non-significant effect of SSG-based training interventions on CODt (ES = 0.16; 95% CI = −0.54 to 0.86; *p* = 0.66; *I*^2^ = 88.2%; Egger's test *p* = 0.274; Figure 7B). The relative weight of each study in the analysis ranged from 31.2 to 36.0%.

### Adverse Effects

Among the included studies, none reported soreness, pain, fatigue, injury, damage, or adverse effects related to the SSG-based and running-based HIIT interventions.

## Discussion

The current SRMA aimed to compare the effects of SSG-based training and running-based HIIT on several neuromuscular-dependent variables (linear sprinting, vertical jumping, and CODt). The comparisons conducted in the meta-analysis revealed a significant favorable effect of running-based HIIT on linear sprinting, while no significant differences were found for vertical jumping and change-of-direction.

### SSGs' vs. Running-Based HIIT Interventions' Effects on Linear Sprinting Time

While there are similarities between SSGs and real games, several factors differentiate them (Clemente, 2020). Determinant external load outcomes, such as high-speed running, sprinting, or accelerations, reveals that SGGs underexpose soccer players to the typical demands of a real game (Gabbett and Mulvey, 2008; Casamichana et al., 2012; Clemente et al., 2019; Dalen et al., 2019). Regarding sprinting, it is necessary to have longitudinal distance as well as opportunities to reach velocity (Nassis et al., 2019) and such fact it is difficult in SSG scenarios in which the pitch is smaller than that of a normal game.

One study revealed that 5 vs. 5, 6 vs. 6, and 9 vs. 9 formats produced, respectively, high-speed running (i.e., running between 19.8 and 25 km/h) mean values of 0, 1.5, and 2.0 m/min, respectively; in official matches, the mean values of the same players was 5 m/min (Clemente et al., 2019). This finding was possibly due to the reduced longitudinal space of the SSG pitch. In the specific case of sprinting (>25 km/h), even greater differences were observed. In 4 vs. 4 and 6 vs. 6 SSG formats, players achieved a mean of 0.2 m/min (vs. 1.7 m/min in an official match) (Dalen et al., 2019). Thus, it is expected that even in larger SSGs (Hill-Haas et al., 2008b), the variability of the games may not expose players to a significant amount of linear sprinting. This, in turn, may have consequences related to the adaptations promoted by SSG-based interventions when compared to running-based interventions (e.g., HIIT) (in the different modes).

The five studies included in this review (Radziminski et al., 2013; Faude et al., 2014; Jastrzebski et al., 2014; Stojiljković et al., 2019; Arslan et al., 2020) include a meta-comparison that revealed a significant favorable effect of running-based HIIT on linear sprinting. Additionally, a within-group analysis revealed a significant positive effect of running-based HIIT and a non-significant effect of SSG interventions.

It is fair to compare the protocols used among the included studies. For example, four of the studies (Faude et al., 2014; Jastrzebski et al., 2014; Stojiljković et al., 2019; Arslan et al., 2020) compared short-interval HIIT (15 s-to-15 s of work-to-rest) with long-interval SSG (2.5 min-to-4 min). Thus, it is admissible that, bioenergetically, the conditions of interventions are not the same. Additionally, SSG formats varied between 2 vs. 2 (Arslan et al., 2020) and 4 vs. 4 (Faude et al., 2014; Stojiljković et al., 2019) with a range of 18- (Radziminski et al., 2013; Jastrzebski et al., 2014) to 40-m length (Faude et al., 2014). Under such conditions, it is realistic to assume that sprinting will not occur very often during SSGs, with natural differences in mechanics and neuromuscular stimulus comparing to running-based HIIT.

Despite those differences in experimental conditions, it is plausible that running-based HIIT (namely, short-interval and more specific modes as repeated sprint training or sprint interval training) can be highly efficient in promoting positive adaptations when compared to SSGs, mainly because of the bioenergetic and mechanical conditions (Buchheit and Laursen, 2013b).

### SSGs' vs. Running-Based HIIT Interventions' Effects on Vertical Height Jump

Vertical jumping performance can be strongly influenced by strength and power, as well as determinant mechanisms such as limb morphology, stiffness, or contractile and elastic elements (Olberding et al., 2019). Different strength and power training methods—namely, the well-known plyometric training (or reactive strength training), which is often used in soccer—have led to positive adaptations (Ramirez-Campillo et al., 2020; van de Hoef et al., 2020). However, sprint and CODt training methods have also shown some positive effects on vertical jumping performance, according to a meta-analysis conducted in soccer (García-Ramos et al., 2018).

In the current SRMA, none of the groups (SSG and running-based HIIT) had significant benefits on jumping performance after interventions. Additionally, no significant differences were found between groups. These findings are reasonable considering that both groups were focused on short- and long-interval running (Faude et al., 2014; Arcos et al., 2015; Stojiljković et al., 2019; Arslan et al., 2020), while none used maximal running methods such as repeated sprint training or sprint interval training (Buchheit and Laursen, 2013a). Even when sprinting training methods are applied, it is not likely to observe improvements other than in neuromuscular-dedicated methods (e.g., plyometric training or power training) considering the type of neuromuscular stimulus or even the direction of force applied (García-Ramos et al., 2018).

Possibly, SSGs and running-based HIIT interventions would both benefit from more specific strength and power training methods to improve jumping performance. Some examples of concurrent training benefits combining running-HIIT and strength/power training on vertical jumping have been reported (Wong et al., 2010; Silva, 2019). Additionally, in a cohort study combining SSG-based periodization and a strength training program, significant positive effects were observed on squat and CMJs (Querido and Clemente, 2020). Thus, SSG-based and running-HIIT might both benefit from more specific strength and power training methods for improving this determinant quality.

### SSGs' vs. Running-Based HIIT Interventions' Effects on Change of Direction Time

Because SSGs are often played on smaller pitches than real games, many quick changes of direction and accelerations/decelerations may occur, both with and without the ball (Clemente, 2020). Although a few specific-oriented interventions have compared SSGs and multidirectional running (in soccer and Australian football), inconsistent findings have been presented (Chaouachi et al., 2014; Young and Rogers, 2014). For example, a study comparing SSG and multidirectional running in soccer revealed significant improvements among a running group on change of direction tests without the ball, while the SSG group exhibited significant improvements on drill-based tests (Chaouachi et al., 2014).

In the current SRMA, a very limited number of studies were included regarding the effects on CODt (Faude et al., 2014; Stojiljković et al., 2019; Arslan et al., 2020). No significant differences were observed between SSGs and running-based HIIT on CODt, but the within-group analysis revealed significant improvements in the running-based HIIT group and no significant changes in SSG-group. One possible explanation for the non-significant effect of SSG is that only COD tests without the ball were included as outcomes. Naturally, this would influence the final result as previously reported (Chaouachi et al., 2014). Moreover, COD, accelerations, and decelerations occurring in SSGs can be highly variable both between players and within players (from repetition to repetition and session to session) (Clemente, 2020). This could affect the consistency of the stimulus on the players.

On the other hand, running-based HIIT generated significant benefits on CODt, perhaps because all the included studies (Faude et al., 2014; Stojiljković et al., 2019; Arslan et al., 2020) used short-interval training with COD, thus eliciting a greater velocity while running with a direct transfer on how the COD is performed (Beato et al., 2019).

### Potential Limitations, Directions for Future Research, and Practical Implications

A possible limitation of this SRMA is that it only included articles written in English published on the Web of Science, Scopus, SPORTDiscus, and PubMed databases. A such, some relevant publications could have been overlooked. Among the revised parallel studies, some methodological limitations were also found. For example, the training interventions did not have similar protocols across studies (e.g., short intervals vs. long intervals), which could have influenced the final results. Additionally, the formats and pitch configurations of the SSGs were very small. As a result, the players did not have longitudinal distances to cover, and this could have affected outcomes related to distance-related qualities (e.g., sprinting). Thus, it is highly recommended that future research include different formats and larger pitches, thus allowing different physical demands to be fairly assessed.

Despite the limitations presented in the included parallel studies, it was observed that SSG-based interventions had no significant effect in any of the outcomes included. Also, running-based HIIT was significantly better than SSG in terms of linear sprinting. This suggests that, despite the apparent positive aspects of SSGs in some respects (e.g., aerobic performance) (Moran et al., 2019), the capacity of SSGs to enhance different physical qualities in soccer is limited (as is true of any training method).

However, this does not mean that SSGs cannot be used. Instead, SSGs could be used in combination with other approaches. For example, combining SSGs and running-based HIIT could be beneficial to soccer players. Two promising original studies using such an approach revealed the positive effects of this combination on aerobic performance (Harrison et al., 2015) and final velocity reached on the 30–15 Intermittent Fitness Test (Rabbani et al., 2019). Thus, in future research, the effects of such a combination on sprinting, jumping, and CODt performance can be compared to the effects of SSGs or running-based HIIT alone.

Additionally, a combination of SSG with strength/power training could represent an interesting solution and could have practical implications considering the promising findings revealed by a cohort study (Querido and Clemente, 2020). Naturally, the experimental protocols and methodological quality of future studies should be improved, by considering bias as concurrent effects of training loads, fitness status or other constraints. Additionally, randomized controlled trials should be used, and the magnitude of changes should be determined using different frequencies, volumes, and intensities of stimuli.

Finally, among the included studies, none were conducted in women or adults (amateurs or professionals). All of them were conducted in youth male soccer players. This represents a glaring gap in the literature that should be progressively corrected. Additionally, as an experimental approach, it is also important to start reporting data for responders and non-responders while also noting the influence of specific factors as baseline levels, maturation status, or load accumulated in a field-based training context.

## Conclusions

This SRMA aimed to compare the effects of SSG and running-based HIIT interventions on linear sprint performance, vertical jump, and change of direction performance. In short, running-based HIIT yielded significantly greater improvements than SSG-based interventions in terms of sprinting performance, while no significant differences were found for jumping performance and CODt. The within-group analysis revealed no significant benefits of SSG-based interventions on any of the outcomes, while running-based HIIT presented significant positive effects for linear sprinting and COD performance.

Despite similarities of SSGs and running-based HIIT in improving aerobic performance, it seems that effects are not the same in powerful actions as sprinting, vertical jump, or COD. Therefore, it may be not prudent to totally replace running-based methods with SSGs. Combining SSG and running-based HIIT may be a possible alternative. Another alternative training method involves implementing strength and power interventions as part of the standard supplementation of SSG-based periodization.

## Data Availability Statement

The original contributions presented in the study are included in the article/Supplementary Material, further inquiries can be directed to the corresponding author/s.

## Author Contributions

FC lead the project, run the data search and methodological assessment, and wrote and revised the original manuscript. RR-C analyzed and interpreted the data, wrote the statistical report, and revised the original manuscript. JA wrote and revised the original manuscript. HS run the data search and methodological assessment and wrote and revised the original manuscript.

## Conflict of Interest

The authors declare that the research was conducted in the absence of any commercial or financial relationships that could be construed as a potential conflict of interest.

## References

[B1] Aquino R. Melli-Neto B. Ferrari J. V. S. Bedo B. L. S. Vieira L. H. P. Santiago P. R. P.. (2019). Validity and reliability of a 6-a-side small-sided game as an indicator of match-related physical performance in elite youth Brazilian soccer players. J. Sports Sci. 37, 2639–2644. 10.1080/02640414.2019.1608895 31064264

[B2] Arcos A. L. Vázquez J. S. Martín J. Lerga J. Sánchez F. Villagra F.. (2015). Effects of small-sided games vs. interval training in aerobic fitness and physical enjoyment in young elite soccer players. PLoS ONE 10:e0137224. 10.1371/journal.pone.0137224 26331623 PMC4558056

[B3] Arslan E. Orer G. Clemente F. (2020). Running-based high-intensity interval training vs. small-sided game training programs: effects on the physical performance, psychophysiological responses and technical skills in young soccer players. Biol. Sport 37, 165–173. 10.5114/biolsport.2020.94237 32508384 PMC7249797

[B4] Beato M. Coratella G. Bianchi M. Costa E. Merlini M. (2019). Short-term repeated-sprint training (straight sprint vs. changes of direction) in soccer players. J. Hum. Kinet. 70, 183–190. 10.2478/hukin-2019-0040 31915488 PMC6942460

[B5] Buchheit M. Laursen P. B. (2013a). High-intensity interval training, solutions to the programming puzzle : part I: cardiopulmonary emphasis. Sport. Med. 43:313–338. 10.1007/s40279-013-0029-x 23539308

[B6] Buchheit M. Laursen P. B. (2013b). High-intensity interval training, solutions to the programming puzzle : part II: anaerobic energy, neuromuscular load and practical applications. Sport. Med. 43, 927–954. 10.1007/s40279-013-0066-5 23832851

[B7] Bujalance-Moreno P. Latorre-Roman P. A. Garcia-Pinillos F. (2019). A systematic review on small-sided games in football players: acute and chronic adaptations. J. Sports Sci. 37, 921–949. 10.1080/02640414.2018.1535821 30373471

[B8] Casamichana D. Castellano J. Castagna C. (2012). Comparing the physical demands of friendly matches and small-sided games in semiprofessional soccer players. J. Strength Cond. Res. 26, 837–843. 10.1519/JSC.0b013e31822a61cf 22310516

[B9] Chaouachi A. Chtara M. Hammami R. Chtara H. Turki O. Castagna C. (2014). Multidirectional sprints and small-sided games training effect on agility and change of direction abilities in youth soccer. J. Strength Cond. Res. 28, 3121–3127. 10.1519/JSC.0000000000000505 25148467

[B10] Clemente F. M. (2020). The threats of small-sided soccer games. Strength Cond. J. 42:1. 10.1519/SSC.0000000000000526

[B11] Clemente F. M. Afonso J. Castillo D. Arcos A. L. Silva A. F. Sarmento H. (2020). The effects of small-sided soccer games on tactical behavior and collective dynamics: a systematic review. Chaos, Solitons and Fractals 134:109710. 10.1016/j.chaos.2020.109710

[B12] Clemente F. M. Ramirez-Campillo R. Nakamura F. Y. Sarmento H. (2021). Effects of high-intensity interval training in men soccer player's physical fitness: a systematic review with meta-analysis of randomized-controlled and non-controlled trials. J. Sports Sci.. 10.1080/02640414.2020.1863644. [Epub ahead of print]. 33423603

[B13] Clemente F. M. Sarmento H. Rabbani A. Van Der Linden C. M. I. N. Kargarfard M. Costa I. T. (2019). Variations of external load variables between medium- and large-sided soccer games in professional players. Res. Sport. Med. 27, 50–59. 10.1080/15438627.2018.1511560 30129780

[B14] Dalen T. Sandmæl S. Stevens T. G. Hjelde G. H. Kjøsnes T. N. Wisløff U. (2019). Differences in acceleration and high-intensity activities between small-sided games and peak periods of official matches in elite soccer players. J. Strength Cond. Res. 10.1519/JSC.0000000000003081. [Epub ahead of print]. 30741867

[B15] Davids K. Araújo D. Correia V. Vilar L. (2013). How small-sided and conditioned games enhance acquisition of movement and decision-making skills. Exerc. Sport Sci. Rev. 41, 154–161. 23558693 10.1097/JES.0b013e318292f3ec

[B16] Deeks J. J. Higgins J. P. Altman D. G. (2008). Analysing data and undertaking meta-analyses, in Cochrane Handbook for Systematic Reviews of Interventions: The Cochrane Collaboration, eds J. P. Higgins and S. Green (The Cochrane Collaboration), 243–296.

[B17] Egger M. Smith G. D. Schneider M. Minder C. (1997). Bias in meta-analysis detected by a simple, graphical test. BMJ 315, 629–634. 10.1136/bmj.315.7109.629 9310563 PMC2127453

[B18] Faude O. Steffen A. Kellmann M. Meyer T. (2014). The effect of short-term interval training during the competitive season on physical fitness and signs of fatigue: a crossover trial in high-level youth football players. Int. J. Sports Physiol. Perform. 9, 936–944. 10.1123/ijspp.2013-0429 24622685

[B19] Gabbett T. J. Mulvey M. J. (2008). Time-motion analysis of small-sided training games and competition in elite women soccer players. J. Strength Cond. Res. 22, 543–552. 10.1519/JSC.0b013e3181635597 18550972

[B20] García-Hermoso A. Ramírez-Campillo R. Izquierdo M. (2019). Is muscular fitness associated with future health benefits in children and adolescents? A systematic review and meta-analysis of longitudinal studies. Sport. Med. 49, 1079–1094. 10.1007/s40279-019-01098-6 30953308

[B21] García-Ramos A. Haff G. G. Feriche B. Jaric S. (2018). Effects of different conditioning programmes on the performance of high-velocity soccer-related tasks: Systematic review and meta-analysis of controlled trials. Int. J. Sports Sci. Coach. 13, 129–151. 10.1177/1747954117711096

[B22] Green S. Higgins J. (2005). Cochrane Handbook for Systematic Reviews of Interventions. The Cochrane Collaboration.31643080

[B23] Group C. C. C. R. (2016). Data Extraction Template for Included Studies. Group C. C. C. R.

[B24] Hammami A. Gabbett T. J. Slimani M. Bouhlel E. (2018). Does small-sided games training improve physical-fitness and specific skills for team sports? A systematic review with meta-analysis. J. Sports Med. Phys. Fitness 58, 1446–1455. 10.23736/S0022-4707.17.07420-5 29072025

[B25] Harrison C. Kinugasa T. Gill N. Kilding A. (2015). Aerobic fitness for young athletes: combining game-based and high-intensity interval training. Int. J. Sports Med. 36, 929–934. 10.1055/s-0034-1396825 26212246

[B26] Higgins J. P. T. (2003). Measuring inconsistency in meta-analyses. BMJ 327, 557–560. 10.1136/bmj.327.7414.557 12958120 PMC192859

[B27] Higgins J. P. T. Thompson S. G. (2002). Quantifying heterogeneity in a meta-analysis. Stat. Med. 21, 1539–1558. 10.1002/sim.1186 12111919

[B28] Hill-Haas S. Coutts A. Rowsell G. Dawson B. (2008a). Variability of acute physiological responses and performance profiles of youth soccer players in small-sided games. J. Sci. Med. Sport 11, 487–490. 10.1016/j.jsams.2007.07.006 17825620

[B29] Hill-Haas S. Rowsell G. Coutts A. Dawson B. (2008b). The reproducibility of physiological responses and performance profiles of youth soccer players in small-sided games. Int. J. Sports Physiol. Perform. 3, 393–396. 10.1123/ijspp.3.3.393 19211950

[B30] Hill-Haas S. V. Dawson B. Impellizzeri F. M. Coutts A. J. (2011). Physiology of small-sided games training in football a systematic review. Sport. Med. 41, 199–220. 10.2165/11539740-000000000-00000 21395363

[B31] Hopkins W. G. Marshall S. W. Batterham A. M. Hanin J. (2009). Progressive statistics for studies in sports medicine and exercise science. Med. Sci. Sport. Exerc. 41, 3–13. 10.1249/MSS.0b013e31818cb278 19092709

[B32] Iaia F. M. Rampinini E. Bangsbo J. (2009). High-intensity training in football. Int. J. Sports Physiol. Perform. 4, 291–306. 10.1123/ijspp.4.3.291 19953818

[B33] Jastrzebski Z. Barnat W. Dargiewicz R. Jaskulska E. Szwarc A. Radzimiński Ł. (2014). Effect of In-season generic and soccer-specific high-intensity interval training in young soccer players. Int. J. Sports Sci. Coach. 9, 1169–1179. 10.1260/1747-9541.9.5.1169

[B34] Jeong T. S. Reilly T. Morton J. Bae S. W. Drust B. (2011). Quantification of the physiological loading of one week of pre-season and one week of in-season training in professional soccer players. J. Sports Sci. 29, 1161–1166. 10.1080/02640414.2011.583671 21777053

[B35] Kontopantelis E. Springate D. A. Reeves D. (2013). A re-analysis of the cochrane library data: the dangers of unobserved heterogeneity in meta-analyses. PLoS ONE 8:e69930. 10.1371/journal.pone.0069930 23922860 PMC3724681

[B36] Kunz P. Engel F. A. Holmberg H.-C. Sperlich B. (2019). A meta-comparison of the effects of high-intensity interval training to those of small-sided games and other training protocols on parameters related to the physiology and performance of youth soccer players. Sport. Med. Open 5:7. 10.1186/s40798-019-0180-5 30790134 PMC6384288

[B37] Moher D. Liberati A. Tetzlaff J. Altman D. G. (2009). Preferred reporting items for systematic reviews and meta-analyses: the PRISMA statement. PLoS Med. 6:e1000097. 10.1371/journal.pmed.1000097 19621072 PMC2707599

[B38] Moran J. Blagrove R. C. Drury B. Fernandes J. F. T. Paxton K. Chaabene H.. (2019). Effects of small-sided games vs. conventional endurance training on endurance performance in male youth soccer players: a meta-analytical comparison. Sport. Med. 49, 731–742. 10.1007/s40279-019-01086-w 30868441

[B39] Moran J. Ramirez-Campillo R. Granacher U. (2018). Effects of jumping exercise on muscular power in older adults: a meta-analysis. Sport. Med. 48, 2843–2857. 10.1007/s40279-018-1002-5 30341594

[B40] Nassis G. P. Brito J. Figueiredo P. Gabbett T. J. (2019). Injury prevention training in football: let's bring it to the real world. Br. J. Sports Med. 10.1136/bjsports-2018-100262. [Epub ahead of print]. 30926629

[B41] Olberding J. P. Deban S. M. Rosario M. V. Azizi E. (2019). Modeling the determinants of mechanical advantage during jumping: consequences for spring- and muscle-driven movement. Integr. Comp. Biol. 59, 1515–1524. 10.1093/icb/icz139 31397849

[B42] Querido S. M. Clemente F. M. (2020). Analyzing the effects of combined small-sided games and strength and power training on the fitness status of under-19 elite football players. J. Sports Med. Phys. Fitness 60, 1–10. 10.23736/S0022-4707.19.09818-9 32008309

[B43] Rabbani A. Clemente F. M. Kargarfard M. Jahangiri S. (2019). Combined small-sided game and high-intensity interval training in soccer players: the effect of exercise order. J. Hum. Kinet. 69, 249–257. 10.2478/hukin-2018-0092 31666907 PMC6815089

[B44] Radziminski L. Rompa P. Barnat W. Dargiewicz R. Jastrzebski Z. (2013). A comparison of the physiological and technical effects of high-intensity running and small-sided games in young soccer players. Int. J. Sports Sci. Coach. 8, 455–466. 10.1260/1747-9541.8.3.455

[B45] Ramirez-Campillo R. Sanchez-Sanchez J. Romero-Moraleda B. Yanci J. García-Hermoso A. Manuel Clemente F. (2020). Effects of plyometric jump training in female soccer player's vertical jump height: A systematic review with meta-analysis. J. Sports Sci. 38, 1475–1487. 10.1080/02640414.2020.1745503 32255389

[B46] Redkva P. E. Paes M. R. Fernandez R. Da-Silva S. G. (2018). Correlation between match performance and field tests in professional soccer players. J. Hum. Kinet. 62, 213–219. 10.1515/hukin-2017-0171 29922392 PMC6006532

[B47] Sarmento H. Clemente F. M. Harper L. D. Costa I. T. da Owen A. Figueiredo A. J. (2018). Small sided games in soccer – a systematic review. Int. J. Perform. Anal. Sport 18, 693–749. 10.1080/24748668.2018.151728833030987

[B48] Silva J. R. (2019). Concurrent aerobic and strength training for performance in soccer, in Concurrent Aerobic and Strength Training (Cham: Springer International Publishing), 397–416. 10.1007/978-3-319-75547-2_27

[B49] Skrede T. Steene-Johannessen J. Anderssen S. A. Resaland G. K. Ekelund U. (2019). The prospective association between objectively measured sedentary time, moderate-to-vigorous physical activity and cardiometabolic risk factors in youth: a systematic review and meta-analysis. Obes. Rev. 20, 55–74. 10.1111/obr.12758 30270500

[B50] Slim K. Nini E. Forestier D. Kwiatkowski F. Panis Y. Chipponi J. (2003). Methodological index for non-randomized studies (MINORS): development and validation of a new instrument. ANZ J. Surg. 73, 712–716. 10.1046/j.1445-2197.2003.02748.x 12956787

[B51] Stevens T. G. A. De Ruiter C. J. Beek P. J. Savelsbergh G. J. P. (2016). Validity and reliability of 6-a-side small-sided game locomotor performance in assessing physical fitness in football players. J. Sports Sci. 34, 527–534. 10.1080/02640414.2015.1116709 26630259

[B52] Stojiljković N. Gušić M. Molnar S. (2019). Small-sided games versus interval training in adolescent soccer players: effects on speed, change of direction speed and jumping performance. Homo Sport. 21, 55–60.

[B53] Stolen T. Chamari K. Castagna C. Wisloff U. (2005). Physiology of soccer: an update. Sport. Med. 35, 501–536. 10.2165/00007256-200535060-00004 15974635

[B54] van de Hoef P. A. Brauers J. J. van Smeden M. Backx F. J. G. Brink M. S. (2020). The effects of lower-extremity plyometric training on soccer-specific outcomes in adult male soccer players: a systematic review and meta-analysis. Int. J. Sports Physiol. Perform. 15, 3–17. 10.1123/ijspp.2019-0565 31810063

[B55] Wong P. Chaouachi A. Chamari K. Dellal A. Wisloff U. (2010). Effect of preseason concurrent muscular strength and high-intensity interval training in professional soccer players. J. Strength Cond. Res. 24, 653–660. 10.1519/JSC.0b013e3181aa36a2 19816215

[B56] Young W. Rogers N. (2014). Effects of small-sided game and change-of-direction training on reactive agility and change-of-direction speed. J. Sports Sci. 32, 307–314. 10.1080/02640414.2013.823230 24016360

